# Transcranial Random Noise Stimulation Does Not Improve Behavioral and Neurophysiological Measures in Patients with Subacute Vegetative-Unresponsive Wakefulness State (VS-UWS)

**DOI:** 10.3389/fnhum.2017.00524

**Published:** 2017-11-06

**Authors:** Mauro Mancuso, Laura Abbruzzese, Stefania Canova, Giulia Landi, Simone Rossi, Emiliano Santarnecchi

**Affiliations:** ^1^Tuscany Rehabilitation Clinic, Montevarchi, Italy; ^2^Siena Brain Investigation and Neuromodulation Lab, Department of Medicine, Surgery and Neuroscience, University of Siena, Siena, Italy; ^3^Berenson-Allen Center for Noninvasive Brain Stimulation, Department of Cognitive Neurology, Harvard Medical School, Boston, MA, United States

**Keywords:** non-invasive brain stimulation, disorders of consciousness, transcranial electric stimulation, tRNS, vegetative state

## Abstract

**Background:** The absence of efficient treatments capable to promote central nervous system recovery in patients in vegetative state (VS) due to a severe acquired brain injury highlights the need of exploring alternative neuromodulatory treatments that can lead to neurobehavioral gains. Some encouraging preliminary observations suggest that transcranial direct current stimulation could be effective in disorders of consciousness (DoC) patients, especially when applied on the dorsolateral prefrontal cortex (DLPFC) in patients with minimally conscious state (MCS) but not in those with VS.

**Objective:** The primary aim of the present study was to verify if the application of transcranial random noise stimulation (tRNS) on the DLPFC might favor improvements of consciousness recovery in subacute VS-UWS.

**Methods:** Nine patients with DoC due to traumatic brain injury (*n* = 1), anoxia (*n* = 3), and vascular damage (*n* = 5), have undergone a randomized, double-blind, sham-controlled, neuromodulatory trial with tRNS of bilateral DLPFC. All patients were in a post-acute phase and the DoC onset ranged from 30 days to 4 months. The diagnosis of DoC was based on internationally established criteria from the Multi-Society Task Force on PVS, and classified as VS or MCS using the JFK Coma Recovery Scale-Revised scores (CRS-R). We used CRS-R, Synek Scale, Ad-Hoc semi-quantitative scale and the Clinical Global Impression-Improvement scale to measure behavioral and electrophysiological changes during tRNS intervention. All patients were also treated with daily conventional rehabilitation treatment.

**Results:** No significant differences emerged between active and sham groups regarding improvements of level of consciousness, as well as on electroencephalographic data. Only one patient showed emergence from VS-UWS, evolving from VS to MCS after the tRNS stimulation, at a distance of 3 weeks from the enrolment into the study.

**Conclusion:** Repeated applications of tRNS of the DLPFC, even if applied in a subacute phase of VS-UWS state, did not modify behavioral and neurophysiological outcomes differently than sham stimulation.

## Introduction

The term vegetative state (VS) refers to patients who have awakened from coma but remain unresponsive, showing wakefulness without awareness (Jennett and Plum, 1972). It was called VS to highlight the preserved vegetative nervous functioning, in terms of preserved sleep-wake cycles, breathing, digestion, or thermoregulation. Jennett and Plum (1972) first coined the term persistent vegetative state (PVS) to denote a condition that remain for at least 1 month after the insult. Although the term PVS is widely used within the medical community and scientific literature, this clinical syndrome was initially termed apallic syndrome (Kretschmer, 1940), or vigilant coma (Calvet and Coll, 1959). The Multi-Society Task Force on PVS (1994) introduced the notion of permanent VS defining the temporal criteria for irreversibility: more than 1 year for traumatic and 3 months for non-traumatic (anoxic) etiology. More recently, the need to overcome the strong negative connotations that the term “PVS” continues to have after over 35 years, led the European Task Force on Disorders of Consciousness to introduce the term of Unresponsive Wakefulness Syndrome (UWS) (2010). As this neutral descriptive term indicates, UWS refers to a clinical syndrome describing patients who fail to exhibited voluntary motor responsiveness to commands in the presence of clinical signs of eyes-open wakefulness (Laureys et al., 2010). This complex syndrome is exerting a heavy impact on the health system and the efforts spent to identify patients that could emerge from this state recovering consciousness (Bruno et al., 2012) did not lead to standardization of evidence-based guidelines for the treatment of this condition (Bernat, 2006). At the moment, all the chronic disorders of consciousness states (DoC) lacks of effective pharmacological treatment options (Gosseries et al., 2011).

Moreover, invasive and non-invasive brain stimulation interventions, such as repetitive transcranial magnetic stimulation (rTMS) (Ragazzoni et al., 2017), have substantially failed to show significantly and reproducible clinical effects (Cincotta et al., 2015). However, although evidence-based data are still lacking (Lefaucheur et al., 2017), some encouraging preliminary observations suggest that neuromodulatory approaches as those involving transcranial direct current stimulation (tDCS) may be beneficial in DoC patients. Studies that used this type of neurostimulation in DoC patients demonstrated that anodal tDCS applied on the dorsolateral prefrontal cortex (DLPFC) may transiently improve signs of consciousness in patients with minimally conscious state (MCS) but not in those with chronic (i.e., more than 1 year from onset) persistent vegetative state-unresponsive wakefulness state (VS-UWS) (Angelakis et al., 2014; Thibaut et al., 2014). Mechanistically, the response to tDCS in MCS patients could be mediated by the supposed effect of tDCS on local DLPFC excitability levels, which is thought to then engage the connectivity between DLPFC and midline cortical structures, including the anterior cingulate cortex and precuneus. Even though tDCS constitute a very promising tool with clinical potential, issues related to the role of the bipolar field induced into the brain (i.e., both anodal and cathodal stimulation are delivered, on different regions, at the same time) remains open and do not help a clear interpretation of the observed effects (Bestmann et al., 2015; Santarnecchi et al., 2015).

A still unexplored neuromodulatory intervention in DoC patients is the application of transcranial random noise stimulation (tRNS) (Terney et al., 2008). High-frequency tRNS is a recently developed form of transcranial electrical stimulation based on the injection of a multi-frequency (between 101 and 640 Hz) electrical oscillatory spectrum in the form of white noise, capable to induce long-lasting effects on cortical excitability when applied on the scalp overlying the motor cortex (Terney et al., 2008) and DLPFC (Snowball et al., 2013). Long-term potentiation (LTP) has been postulated as a likely mechanism underlying these after-effects (Nitsche et al., 2009), with Terney et al. (2008) demonstrating effects on both cortical excitability and behavior (motor learning). It has been demonstrated that tRNS exerts more gradual effects respect to tDCS and that ideal timing for tRNS application is during task execution (i.e., online effect) (Pirulli et al., 2013). The after-effect of tRNS is intensity dependent. Lower intensity stimulation of about 0.4 mA leads to inhibitory after-effects comparable to what has been observed with cathodal tDCS using 1 mA or 140 Hz tACS at 0.4 mA (Moliadze et al., 2012). Recently, a significant improvement during a visual perceptual learning task was found by Fertonani et al. (2011) by applying high-frequency tRNS on the visual cortex of healthy subjects, as compared to anodal tDCS; also, tRNS over the lateral occipital cortex facilitated facial identity perception in a similar study on healthy participants (Romanska et al., 2015). The effects of tRNS has been explained in the context of the stochastic resonance phenomenon, according to which tRNS might induce random activity in the affected neuronal populations, thus promoting the sensitivity of the neurons to a given range of weak inputs and thereby increasing the signal-to-noise ratio (Stacey and Durand, 2000). An alternative explanation sees tRNS preventing the homeostasis of the system through repeated subthreshold stimulations (Fertonani et al., 2011).

Despite its potential, tRNS has not yet tested as a neuromodulatory intervention in DoC patients. Therefore, in the present study we explored the possibility of inducing clinically relevant effects in a population of VS-UWS patients by means of tRNS. Differently from all previous clinical trials using non-invasive neuromodulation in chronic (i.e., after 6 months from the initial event) MCS or VS-UWS patients (Ragazzoni et al., 2017), here we performed the neuromodulatory intervention in the acute phase of the DoC (i.e., within 2 months from the onset). We reasoned that favoring plasticity with tRNS, in parallel with usual daily rehabilitation treatment, might have more chance of success in the acute phase rather than in a chronic stage, when the recovery is less likely to occur (Bagnato et al., 2013). As stimulation site, we choose the DLPFC, according to previous encouraging results with tDCS in MCS patients (Angelakis et al., 2014; Thibaut et al., 2014).

The rationale was to activate the DLPFC to improve cognitive function, in line with the known role of the lateral fronto-parietal cortical areas in external (environmental) awareness (Van Haudenhuyse et al., 2011) as compared to midline cortical areas (precuneus, anterior and posterior cingulated cortex, mesio-frontal-parahippocampal areas) involved in internal awareness (Ambrus et al., 2010; Angelakis et al., 2014). We hypothesized that tRNS could modulate the functional communication in cortical areas involved in external awareness in VS-UWS patients, by adding noise to neural oscillations according with the above mentioned stochastic resonance hypothesis (Stacey and Durand, 2000).

Therefore, the primary aim of the present study was to verify if the application of tRNS might favor the consciousness recovery in patients with VS-UWS in acute phase (Laureys et al., 2010). Secondary aim was to verify whether tRNS induced some detectable changes on electroencephalographic (EEG) brain activity, eventually associated with changes in the level of consciousness.

## Materials and Methods

### Methods

#### Patients

Nine patients (seven woman) with DoC were included in a perspective, randomized, double blind, sham controlled, no profit pilot study. The age range was 52–86 years (mean ± SD, 71,7 ± 10 years), and the etiology was traumatic brain injury (TBI) (*n* = 1), anoxia (*n* = 3), and vascular (*n* = 5). The length of VS-UWS state ranged from 30 days to 4 months (mean ± SD, 45 ± 31.8 days). All patients had been classified as VS-UWS based on JFK Coma Recovery Scale-Revised (CRS-R) and on internationally established criteria (The Multi-Society Task Force on PVS, 1994). All patients fulfilled the following diagnostic criteria: (a) no evidence of awareness of self or environment and an inability to interact with others; (b) no evidence of sustained, reproducible, purposeful, or voluntary behavioral responses to visual, auditory, tactile or noxious stimuli; no evidence of language comprehension or expression; (c) intermittent wakefulness; (d) bowel and bladder incontinence.

Patients in coma or with a metallic cerebral implant or pacemaker were excluded from the study according to the safety criteria for tDCS in humans. Medications, physiotherapy, and rehabilitation were kept unchanged throughout the trial (see **Table 1** for patients’ characteristics). Rehabilitation treatment consisted of passive therapeutic exercises or mobilization of joint and music therapy for 15 min. The study was conducted according to the Declaration of Helsinki and approved by the local ethics committee of the Siena Health Authority. Written informed consent was obtained from each patient’s legal surrogate.

**Table 1:** Clinical data.

					CRS						
							
	Age	Sex	Etiology	Days from injury	T-1					Therapy	Neuroradioligical data
							
					Day 1	Day 2	Day 3	Day 4	Day 5		
DSO	86	F	Anoxic	122	6	6	6	3	6	BACL	Hypodensity of the periventricular white matter and semioval centers
DR	52	F	Vascular	35	3	6	5	6	6	LEV AMANT	Intra-cerebral hematoma of left basalnucleus
PD	71	F	Vascular	24	6	7	7	7	6	AML AMANT	Right talamo-mesencefalic hemorrhage with ventricular blood flood
SJJ	69	M	Anoxic	27	4	5	4	5	5	LEV AML CLON	Periventricular hypodensity of the white matter due to chronic vascular ischemic suffering
FBL	75	F	Vascular	56	4	4	6	6	6	AML AMANT	Left bilateral cerebellar and occipital hypodensity
MG	64	M	Vascular	38	5	5	6	6	6	AML PHB AMANT LEV	Left supratentorial hemispheric hemorrhagic focus
FM	80	F	Traumatic	23	2	5	5	4	5	AML AMANT LEV	Left acute under-tentorialfronto-parieto-temporal hematoma with associated homolateral frontal subarachnoid intraparenchymal bleeding
SA	69	F	Vascular	59	5	5	5	7	7	VPA AML	Intraparenchymal hematoma due to cerebral arteriovenous malformation with right occipital nodus, right PICA aneurysm
BO	79	M	Anoxic	24	4	4	4	4	4	AMANT	Bilateral hypodensity of the capsular nucleus
Mean	71,7 (52–86)			45,3 (23–122)	4,3	5,2	5,3	5,6	5,9		


*Demographic, MRI as well as clinical scores are reported for each patient. CRS-R, Coma Recovery Scale-Revised; LEV, leviracetam; CLON, clonidina; BACL, baclofene; PHB, phenobarbital; VPA, valproic acid; AML, amlodipina; AMANT, amantadina; VS, vegetative state.*

### Materials

In order to measure and monitor behavioral and electrophysiological changes throughout the trial, changes in (i) the total score of the CRS-R (Giacino et al., 2004), (ii) scores at the six CRS-R subscales addressing auditory, visual, motor, oromotor/verbal, communication, and arousal processes, (iii) changes in EEG activity and (iv) at the Clinical Global Impression-Improvement (CGI-I) scale (Guy, 1976), were measured as compared to baseline assessment.

#### Coma Recovery Scale-Revised

To verify VS-UWS diagnostic criteria, a standardized clinical evaluation through the Italian Version of JFK CRS-R was performed. The CRS-R is clinically used for characterizing the level of consciousness and for monitoring recovery of neurobehavioral functions in patients with DoC. It consists of 29 hierarchically organized items divided into 6 subscales addressing auditory, visual, motor, oromotor, communication, and arousal processes. The score in each CRS-R item is based on the presence or absence of specific behavioral responses to sensory stimuli (higher score indicates better level of consciousness). At the evaluation prior the inclusion into the study, a (i) score equal to two or less on the auditory, motor, and oromotor/verbal subscales, (ii) equal or less to one on the visual subscale, and (iii) equal to 0 on the communication subscale was seen in each patient (**Table 1**), consistently with the diagnosis of VS-UWS (Giacino et al., 2004).

#### Clinical Global Impressions Scale (CGI)

The Clinical Global Impressions Scale (CGI) is a common applied research tool, useful to identify patient progression and treatment response over time. It consists of a seven-point Likert scale (i.e., ‘much improved’; ‘improved’; ‘minimally improved’; ‘no change’; ‘minimally worse’; ‘worse’; or ‘much worse’) in which the evaluator is requested to assess how much the patient has improved or worsened compared to a baseline assessment (Guy, 1976).

#### Electroencephalography

The EEG was analyzed offline using the Synek scale (Synek, 1988) and an ad hoc semi-quantitative scale (see Supplementary Table S1). The Synek scale allows to classify EEG abnormalities in five different grades ranging from 1 (= dominant alpha activity with some scattered theta activity) to 5 (= isoelectric activity). The ad hoc semi-quantitative scale has been used in a previous study (Cincotta et al., 2015) in order to capture subtle changes in EEG which might be of clinical relevance in DoC patients. At each time point, the presence and the symmetry of the following EEG characteristics has been evaluated: (i) posterior alpha rhythm; (ii) focal and widespread voltage reduction, (iii) paroxysmal activity, (iv) slow activity (delta and theta), (v) fast rhythms; (vi) spontaneous variability; (vii) reactivity to acoustic and painful stimulation.

### Brain Stimulation Conditions and Procedures

All participants underwent randomized tRNS or sham tRNS (S-tRNS) over the left and right DLPFC for 5 consecutive days, immediately before a daily rehabilitative treatment session. Each patient received actual or sham tRNS stimulations in randomized order. tRNS was delivered using a battery driven, wireless, hybrid EEG/tCS eight-channel neurostimulator system (Starstim stimulator, Neuroelectrics), through PISTIM electrodes. Each daily session of stimulation consisted in 20 min of high frequency noise (101–640 Hz) with an intensity of 2 mA and a ramp up/down of 30 s at the beginning and at the end of stimulation. For the sham condition, the same electrode placement was used, but the current was applied for 30 s, and was then ramped down to 0 mA. tRNS was applied over the left and right DLPFC (regions F3 and F4 identified using the standard international 10–20 EEG electrode placement system).

**FIGURE 1:**
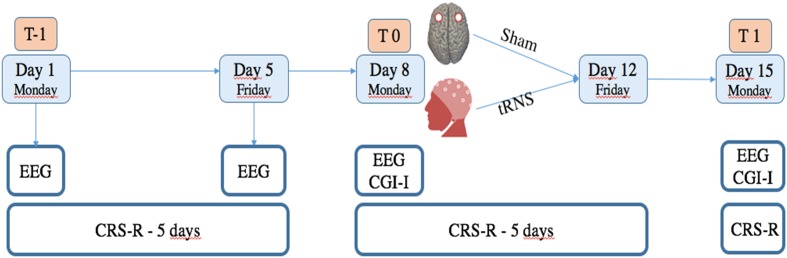
Study design. T-1 corresponds to baseline evaluations in which CRS-R was administered each day (for 5 consecutive days), and 19 channel EEG registration was obtained the first day of enrolment. T0 corresponds to CRS-R, EEG and CGI-I, administered at the beginning of treatment. At T1, evaluation by means of CRS-R, EEG, and CGI-I was repeated to verify effects of treatment.

Patients were assessed with the CRS-R a total of 12 times, including a baseline assessment each day of the week. Specifically: before the treatment (six times between T-1 and T0), during the treatment (five times between T0 and T1, after each day of treatment) and at day 15 (the end of the treatment: T1) (see **Figure 1**). tRNS treatment effect was assessed by means of standardized CRS-R assessments performed by trained and experienced blinded assessors.

Oscillatory brain activity assessment included 40 min of 19-channel electroencephalographic recordings (high-pass filter 0.53 Hz, low-pass filter 30 Hz), carried out at the same time points as the administration of the CRS-R, obtained by means of scalp electrodes placed according to the International 10–20 System (Fp1, Fp2, F7, F3, Fz, F4, F8, T3, C3, Cz, C4, T4, T5, P3, Pz, P4, T6, O1, O2; Nihon Kolden equipment).

At the beginning and at the end of the interventions, the CGI-I scale was performed by a neurologist and also administered to patient’s relatives (Cincotta et al., 2015). The evaluator and relatives were blind to the type of stimulation, as well as to the results of the EEG and clinical evaluations.

The experimenter who performed the clinical evaluations differed from the experimenter who delivered stimulation and was blind to the type (real or sham) of tRNS application as well as to the EEG findings. Moreover, the experimenter who evaluated the EEG differed from experimenters who performed stimulation or clinical assessment and was blind to the type of tRNS application as well as to the clinical findings.

### Statistical Analysis

Mann–Whitney *U*-tests has been used to test the effect of treatment (real tRNS vs. sham) at each time point on the CRS-R total score (T-1, T0, T1) and on scores of the six CRS-R subscales. Differences between treatments were also separately evaluated using repeated-measures ANOVA for the total and subscale CRS-R scores.

Electroencephalographic classification by Synek scale in tRNS and sham stimulation conditions at each time point have been compared by the non-parametric Mann–Whitney *U*-test for independent samples. Moreover, *ad hoc* semi-quantitative scale assessment was analyzed through *F*-Fisher’s test in order to compare the two conditions (real tRNS and sham) at each time point.

Scores of the CGI-I obtained by the neurologist and scores of the CGI-I obtained by the patients’ relatives after the end of intervention (T1) were analyzed by the non-parametric Mann–Whitney *U*-test for independent samples in order to compare real tRNS and sham stimulation conditions. Moreover, the number of patients classified as unchanged/worsened and those classified as improved by the patients’ relatives and by neurologist has been compared using the chi-square test separately for real tRNS and sham stimulation conditions. Finally, the agreement between CGI-I evaluation performed by the patients’ relatives and the neurologist was measured through Cohen’s kappa statistic. Significance was set at *p* < 0.05 for all tests.

## Results

### Randomization and Adverse Effects

Five patients received real tRNS and four patients received sham stimulation. No detectable side effect, neither seizures, occurred in any of the participants.

### Coma Recovery Scale-Revised

**FIGURE 2:**
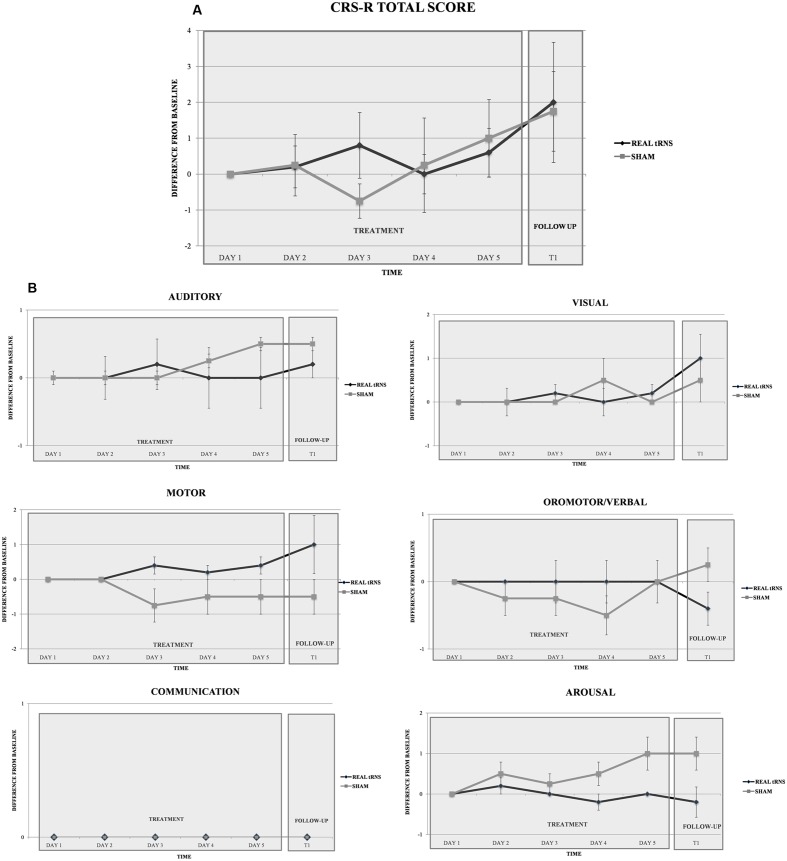
Clinical effect of tRNS. Performance of the two groups (real tRNS and sham) in the total CRS-R scores **(A)** and in the six CRS-R **(B)** subscales scores (expressed in terms of difference from baseline) at different time points.

Repeated-measure ANOVA did not reveal significant differences between real or sham tRNS conditions for the total CRS-R scores [Time: *F*(5,35) = 2.147, *p* = 0.83; Group: *F*(1,7) = 1.231, *p* = 0.304; Interaction time × group: *F*(1,7) = 0.015, *p* = 0.906; CSR-R at each time point: (T0a: *Z* = 0.00, *p* = 1; T0b: *Z* = -0.125, *p* = 0.901; T0c: *Z* = -1.019, *p* = 0.308; T0d: *Z* = -0.254, *p* = 0.8; T0e: *Z* = -0.496, *p* = 0.62; T1 = -0.246, *p* = 0.806)] (**Figure 2**), and also for the CRS subscores [all *p* > 0.05; CSR-R subscales values at each time point: (T0a: *Z* = -0.894, *p* = 0.371; T0b: *Z* = 1.118, *p* = 0.264; T0c: *Z* = -1.757, *p* = 0.079; T0d: *Z* = -2.196, *p* = 0.028; T1: *Z* = 1.783, *p* = 0.075)]. Notably, CSR-R arousal subscale displayed a non-significant trend toward increased arousal at day four of treatment, with higher scores after sham stimulation (**Figure 2**).

### Clinical Global Impression-Improvement

No significant differences emerged between real or sham stimulation conditions in CGI-I scores when the evaluation was performed by the neurologist (T1: *Z* = 0.295). Likewise, no significant CGI-I scores difference between real and sham stimulation when the evaluation was performed by the patients’ relatives (*Z* = 0.441).

**FIGURE 3:**
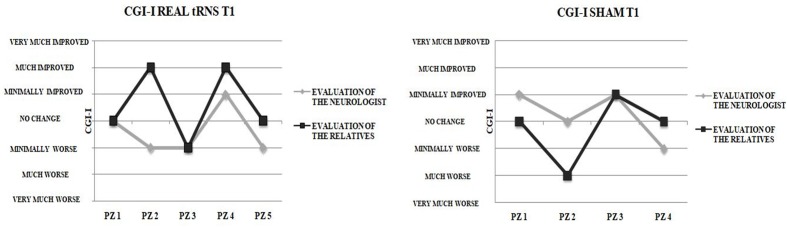
Clinical vs. Relatives evaluation of improvement. Clinical Global Impression-Improvement (CGI-I) evaluations after the end of the 5-days treatment for real tRNS and sham treatment. No concordance was seen between the evaluations performed by the neurologist and the patient’s relatives.

The number of patients classified by neurologist and by patients’ relatives according to CGI-I levels did not differ significantly either for real tRNS and sham stimulation conditions (*p* > 0.05). Notably, poor agreement emerged between the CGI-I evaluations performed by the patients’ relatives after the end of the 5-day interventions and those performed by the neurologist (**Figure 3**), as indicated by means of Cohen’s kappa coefficient either for real tRNS (κ = 0.25, *p* = 0.149) and sham stimulation conditions (κ = 0.00, *p* = 1).

### Electroencephalography

At all the time points, no significant differences emerged between real or sham stimulation in the semi-quantitative EEG parameters evaluated by the Synek scale and by the ad hoc semi-quantitative scale (*p* > 0.05 for all comparisons). After the end of the 5 days of treatment (T1), the EEG activity has been classified from Delta to Theta in 40% of patients that received real tRNS and in 50% of sham condition patients.

No significant differences emerged in the number of patients classified according to Synek scale levels either for real tRNS and sham stimulation conditions (*p* > 0.05 for all comparisons), as shown in **Table 2**.

**Table 2:** Patients EEG profile.

	T-1		T0		T1	
			
	Real tRNS	Sham	Real tRNS	Sham	Real tRNS	Sham
Regular alpha	0	0	0	0	0	0
Predominant theta	3	0	3	1	3	2
Delta/spindles	2	4	2	3	2	2
Burst suppression	0	0	0	0	0	0
Suppression	0	0	0	0	0	0


*Number of patients classified according to levels identified by Synek classification in the two conditions (real tRNS and sham).*

## Discussion

In the current study, 1-week of tRNS over bilateral DLPFC combined with physical/sensory stimulation rehabilitative treatment did not alter the spontaneous clinical course of acute patients with VS-UWS syndrome significantly more than placebo (sham) stimulation. At present, this is the first study in which high frequency tRNS was applied for 5 days in sub-acute VS-UWS patients. The absence of available central nervous system treatments for VS-UWS or MCS patients, apart from the surgical insertion of an intrathecal baclofen pump or peripheral treatments (e.g., physical therapy, speech therapy) in addition to any drugs administered for controlling seizures, highlights the need for novel neuromodulatory treatments that can lead to neurobehavioral gains.

At a descriptive level, only one patient (n. 6) showed clinical improvement right after multiday tRNS (T1), emerging from VS-UWS at a distance of 3 weeks from the beginning of the enrolment in the study. However, no clear neurophysiological or clinical parameter that can at least partly explain his recovery of consciousness were identified. In particular, this patient was a 64 years old male, and he was affected by a stroke that provoked a huge ischemic area involving the temporal lobe in the left hemisphere and the pons. He was enrolled in the study 38 days from onset and previous evaluations with CRS-R conducted the days before the first tRNS session did not show any improvement. The Synek Scale was similar to those of other patients. We did not observe substantial differences in drug treatment compared to other patients enrolled in the study (see **Table 1** for more details). During hospitalization, the patient had pulmonary and urinary infections treated with antibiotics therapy.

This highlights the need for carefully describing the clinical course of individual cases in tCS studies on DoC patients, in order to get a real insight in the potential effect of stimulation. Emergence from VS-UWS perhaps indicates a residual capacity for neural plasticity, prompted by rehabilitative treatment and/or the brain stimulation (Bagnato et al., 2013). Future clinical trials should employ tRNS possibly in comparison with other non-invasive electrical stimulation methods that have provided some beneficial effects in MCS patients (Thibaut et al., 2014). EEG findings did not differ between active and sham tRNS, and, on average, the oscillatory activity slightly improved along the trial course, passing from delta to theta activity in about half of the sample, in both stimulation groups. This data should be interpreted taking into account that the reliability of resting-state paradigms to assess level of consciousness is still a matter of debate (Kondziella et al., 2016). Previous data showed that true incidence of consciousness in patients fulfilling the clinical criteria for VS, as detected by “active” paradigms, is between 5 and 15%, due to low sensitivity of such paradigms. Although a more dominant theta activity usually prevails in MCS (Schiff et al., 2014), we did not note, through CRS-R, any clinical change suggesting emergence from VS-UWS to MCS in these patients. Thus, the transition from a prevailing delta to theta activity likely reflected a gradual improvement of brain functional organization (Schiff et al., 2014), however, unrelated to the neuromodulatory intervention and not sufficient to meaningfully affect the behavioral state.

Despite the novelty of the implemented acute intervention which has been never attempted in previous neuromodulatory trials (Ragazzoni et al., 2017), the present –substantially negative– findings, may depend on several, not mutually exclusive, factors: first, patients were in a VS-UWS state in which, unlike MCS, a breakdown of cortico-cortical and cortico-subcortical functional communication is present (Rosanova et al., 2012; Ragazzoni et al., 2013; Casarotto et al., 2016). Stimulation of the DLPFC, that produced behaviorally detectable effects even after a single tDCS session in MCS patients (Thibaut et al., 2014) when targeting left DLPFC, might have failed just because of a lack of viable neural networks due to the underlying cortical lesions (i.e., lack of connectivity, see **Table 1**). Second, studies in healthy subjects suggest that, despite its lasting behavioral effects even when applied outside the motor cortex (Snowball et al., 2013), tRNS facilitates task performance mostly when applied during task execution (Pirulli et al., 2013). Given the current evidence about a null effect for tRNS when applied before rehabilitative/sensory stimulation interventions future studies should investigate the impact of online tRNS applied in combination with other available therapies.

Clearly, a limitation of the present study is represented by the small sample size. Even more important, the lack of MRI based mapping of the stimulated area should be considered, which might be extremely important given the potential presence of focal brain damage, atrophy, and injury-induced differences in brain topography in our patients. Future studies could employ patient-tailored MRI-guided transcranial electrical stimulations, as well as the additional use of longitudinal functional MRI acquisitions to document possible tRNS-specific changes in functional connectivity.

Moreover, the assessment of the complexity of brain’s response to Transcranial Magnetic Stimulation using EEG represents a promising new tool for detection of consciousness. The perturbational complexity index (PCI) by Casali et al. (2013) quantifies the amount of information (i.e., spectral content of brain EEG signals) and the integration of overall cortico-thalamic system output (i.e., spatial extent of brain activations). Future assessment of VS-UWS patients treated with neuromodulatory interventions (e.g., tDCS, tRNS) might benefit from the implementation of TMS-EEG measures aimed to improve investigation of consciousness in non-communicating patients.

## Conclusion

The current pilot study originally tested the efficacy of tRNS in the subacute phase of VS-UWS, reporting no evident benefit when targeting bilateral DLPFC for 5 consecutive days. Along with evidence from other controlled trials on MCS patients (Ragazzoni et al., 2017), the present data might help the design of future non-invasive neuromodulatory interventions in DoC.

## Ethics Statement

The study was carried out in accordance with the recommendations of Comitato Etico Regione Toscana, Area Vasta Sud-Est, with written informed consent, according with the Declaration of Helsinki, from all legal representative of patients. The protocol was approved by the Ethic Committee of Regione Toscana, Area Vasta Sud-Est.

## Author Contributions

MM, SR, and ES designed the experiment; LA, SC, and GL collected and analyzed the data; SR, LA, and GL wrote the paper; MM, ES, and SR provided critical feedback and gave final approval of manuscript.

## Conflict of Interest Statement

The authors declare that the research was conducted in the absence of any commercial or financial relationships that could be construed as a potential conflict of interest.
