# Supplementary material for: Transcranial Random Noise Stimulation Does Not Improve Behavioral and Neurophysiological Measures in Patients with Subacute Vegetative-Unresponsive Wakefulness State (VS-UWS)
Source: Front Hum Neurosci. 2017 Nov 6;11:524. doi: 10.3389/fnhum.2017.00524 (PMC5681535; doi:10.3389/fnhum.2017.00524)
Supplement: Supplementary file 1 [file Table_1.DOCX]

| ***Ad hoc* EEG scale** | | | | | |
| --- | --- | --- | --- | --- | --- |
|  | **ABSENT** | **PRESENT** | |  |  |
| Posterior alpha rhythm |  |  | |  |  |
| Focal voltage reduction |  |  | | **Region(s):______________** | |
| Widespread voltage reduction |  | Symmetric | Asymmetric | **Most affected side:_______** | |
| Spontaneous variability |  |  | |  |  |
| Reactivity to acoustic stimulation |  |  | |  |  |
| Reactivity to painful stimulation |  |  | |  |  |
| Focal paroxysmal activity |  |  | | **Region(s):______________** | |
| Widespread paroxysmal activity |  | Symmetric | Asymmetric | **Most affected side:_______** | |
| Focal slow activity (delta) |  |  | | **Region(s):______________** | |
| Widespread slow activity (delta) |  | Symmetric | Asymmetric | **Most affected side:_______** | |
| Focal slow activity (theta) |  |  | | **Region(s):______________** | |
| Widespread slow activity (theta) |  | Symmetric | Asymmetric | **Most affected side:_______** | |
| Focal fast rhythms |  |  | | **Region(s):______________** | |
| Widespread fast rhythms |  | Symmetric | Asymmetric | **Most affected side:_______** | |

Table S1 Semi-quantitative scale used to qualify EEG activity at different timepoint (T-1, T0, T1).
